# Blood-based biomarkers suggest prolonged axonal Injury following pediatric mild traumatic brain injury

**DOI:** 10.1038/s41598-024-84053-4

**Published:** 2025-02-04

**Authors:** Andrew R. Mayer, Tracey V. Wick, Jessica R. McQuaid, Masen L. Boucher, Andrew B. Dodd, Cidney R. Robertson-Benta, Harm J. van der Horn, Erik B. Erhardt, Robert E. Sapien, Rawan Tarawneh, Rebekah Mannix

**Affiliations:** 1 The Mind Research Network/Lovelace Biomedical and Environmental Research Institute, Albuquerque, NM 87106 USA; 2 Departments of Psychiatry and Behavioral Sciences, University of New Mexico, Albuquerque, NM 87131 USA; 3 Division of Psychology, University of New Mexico, Albuquerque, NM 87131 USA; 4 Department of Neurology, University of New Mexico, Albuquerque, NM, 87131 USA; 5 Division of Emergency Medicine, Boston Children’s Hospital, Boston, MA USA; 6 Department of Neurology, University of Groningen, University Medical Center Groningen, Groningen, The Netherlands; 7 Department of Math and Statistics, University of New Mexico, Albuquerque, NM 87131 USA; 8 Departments of Emergency Medicine, University of New Mexico, Albuquerque, NM 87131 USA; 9 The Mind Research Network, Pete & Nancy Domenici Hall , Albuquerque, 1101 Yale Blvd. NE, NM 87106 USA

**Keywords:** Blood-based biomarkers, Pediatric mild traumatic brain injury, Concussion, Recovery, Trauma, Diagnostic markers

## Abstract

Pediatric mild traumatic brain injury (pmTBI) affects millions of youth annually but underlying pathophysiology and time for physiological recovery remains unknown. Non-fasting plasma samples were obtained in 59 pmTBI (28 females; age 14.9 ± 2.7) at approximately 7 days and 4 months post-injury and in 41 matched healthy controls (HC: 20 females; age 14.3 ± 2.8). Samples were analyzed for GFAP, NFL, Tau, pTau181 and UCH-L1 protein concentrations in conjunction with a clinical battery. Significant effects of diagnosis (pmTBI > HC) existed at ~ 7 days (*p <* 0.001; Cohen’s d = 0.72) and ~ 4 months (*p =* 0.015; Cohen’s d = 0.41) post-injury for NFL. NFL was also elevated in pmTBI with significant alterations to mental status (e.g., post-traumatic amnesia) relative to patients without (*p =* 0.014; Cohen’s d = 0.77). UCH-L1, GFAP and pTau181 did not differ between groups, but demonstrated negative associations with days post-injury (small to medium effect sizes) suggestive of a more rapid release/clearance. Post-concussive symptoms had the best diagnostic classification accuracy at ~ 7 days, but NFL ranked higher at 4 months post-injury. Preliminary findings highlight dynamic fluctuations in blood-based biomarkers in the first week of pmTBI, with ongoing evidence of protein release (NFL) at 4 months. NFL demonstrated additional promise for delineating injury severity within the spectrum of pmTBI.

**Supplementary Information:**

The online version contains supplementary material available at 10.1038/s41598-024-84053-4.

## Introduction

Pediatric mild traumatic brain injury (pmTBI; used synonymously with concussion) affects approximately 750,000 youth annually in the United States alone^1 ^and may alter neurodevelopmental trajectories for a minority of injured patients^2–4^. The current nosology for classification of injury severity (i.e., mild versus moderate versus severe TBI) is imprecise and subjective, and frequently leads to diagnostic and prognostic misconceptions both within and outside of the medical community^5,6^. For example, large discrepancies in classification rates exist when diagnostic criteria from various expert consensus panels are applied to the same pmTBI sample^7^. Moreover, similar disparate results are obtained when attempting to characterize symptom burden based on self-report using for prognostic purposes^8^. Thus, there is a great need for more objective markers of injury severity and prognosis.

Preclinical data suggest that brain injuries result in multifaceted cellular pathologies, with individual pathological substrates demonstrating different temporal trajectories for return to baseline levels^9,10^. The release of various trauma-related proteins into the bloodstream has emerged as a promising technique for objectively determining the presence of TBI as well as tracking injury progression in vivo^11,12^. Blood-based biomarkers have additionally demonstrated clinical sensitivity and specificity for parsing the spectrum of adult mTBI as indexed by both the presence of structural lesions^13 ^as well as more severe alterations in mental status (i.e., presence of post-traumatic amnesia and/or loss of consciousness; PTA/LOC)^14–16^. However, there is also increasing evidence of potential mismatches between symptomatic and physiological recovery^2,17,18^. Specifically, several adult mTBI studies have reported that certain protein concentration levels (neurofilament light; NFL) may remain increased up to one-month post-injury^16,19^.

Blood protein concentration levels have been shown to vary substantially in both pediatric and geriatric samples relative to adults in large N reference studies^20^, highlighting the need for pediatric-focused studies. To date, the majority of pmTBI studies have focused on the diagnostic sensitivity/specificity of a few select blood-based biomarkers (i.e., glial fibrillary acidic protein [GFAP], ubiquitin c-terminal hydrolase L1 [UCH-L1] and S100 calcium-binding protein B [S100B]) at the acute (e.g., less than 72 h) injury phase^18,21^. Sparser data exists for prognostic indications, or longitudinal follow-up weeks to months post-injury^18,21^. A recent study suggested decreased total tau for pmTBI relative to a healthy control (HC) group within one week of injury, with no changes in NFL^22^. Neither of these biomarkers was altered at six months post-injury (pmTBI *N*= 18), and there were no associations with symptom burden^22^. Finally, results from more severely injured adult samples suggest enhanced diagnostic/prognostic sensitivity of phosphorylated-tau (pTau) variants in comparison to total tau and other (e.g., GFAP, UCH-L1) blood biomarkers^23,24^. Similar work with pTau variants has yet to be performed in pmTBI samples.

The first objective of the current study was therefore to determine if there would be differences in blood protein concentration levels approximately 7 days or 4 months post-injury between pmTBI (*N* = 59) and age- and sex-matched HC (*N* = 41). Our second objective was to use supervised machine learning techniques (Random forests) to examine the diagnostic classification accuracy of these biomarkers, as well as generalized linear models to examine relationships with various injury severity metrics. It was hypothesized that NFL, total tau, and pTau-181 would show group differences at ~ 7 days post-injury, but would return to normal levels by ~ 4 months. GFAP, total tau and UCH-L1 were also predicted to exhibit different temporal trajectories (negative association with days post-injury) relative to NFL (positive association with days post-injury), indicative of a more rapid versus delayed release and clearance from the bloodstream. Finally, we predicted that GFAP, UCH-L1, and NFL levels would differ as a function of traditional measures of injury severity (LOC/PTA)^14,15^, but that none of the biomarkers would be associated with post-concussive symptom burden.

## Materials and methods

### Participants

Patients with pmTBI (8–18 years old) were consecutively recruited from two separate Emergency Department and Urgent Care facilities in the greater Albuquerque area during this prospective cohort design. Inclusion criteria for pmTBI were based on a mixture of the American Congress of Rehabilitation Medicine (upper injury limit) and the Zurich Concussion in Sport Group (lower injury limit). Criteria for pmTBI included a closed head injury with a Glasgow Coma Score ≥ 13, LOC (if present) limited to less than 30 min, PTA (if present) limited to less than 24 h, an alteration in mental status, or at least two new acute symptoms (Zurich criteria).

The maximum enrollment window for pmTBI into the study was 10 days post-injury. The first visit (V1) occurred approximately 1 week post-injury (range = 0–10 days), with a second visit (V2) approximately 4 months later. These time intervals were selected to correspond to assessment periods when a majority of participants were expected to be symptomatic (V1) versus clinically recovered (V2). HC (inclusion criteria 8–18 years old) were recruited from the community via word of mouth or fliers (posted in schools, community billboards, hospital sites, etc.). HC were evaluated at similar intervals to control for neurodevelopment and practice effects associated with repeat testing.

General exclusion criteria for both cohorts included: (1) all major neurological diagnoses (e.g., epilepsy, cerebral palsy), (2) previous TBI with loss of consciousness > 30 min, (3) developmental disorder (autism spectrum disorder or intellectual disability), (4) any medically diagnosed psychiatric disorders other than adjustment disorder, (5) non-English speaking or (6) substance abuse/dependence as confirmed by urine screen (conducted at both visits). Specifically, the urine screen examined for the presence of amphetamines, cocaine, marijuana, methamphetamines, opiates, phencyclidine, benzodiazepines, barbiturates, methadone, and methyl​enedioxy-​methamphetamine. In addition, HC with a history of a recent mTBI (within last 6 months) or pmTBI with a history of additional concussive events within the last 6 months were also excluded.

Pediatric mTBI were additionally excluded if they received general anesthesia or required surgery as part of routine care for their injury. HC were additionally excluded if diagnosed with Attention Deficit Hyperactive Disorder or a learning disability.

## Standard protocol approvals, registrations, and patient consents

All procedures were performed in accordance with the ethical standards of the 1964 Helsinki Declaration and its later amendments. The study was further approved by the University of New Mexico School of Medicine Institutional Review Board. All participants provided written informed consent (age of 18 years old) or assent (age of less than 18 years) depending on age at enrollment, with parental consent also obtained for everyone under 18 years of age. All study procedures were performed in person at an outpatient research assessment center. The first author (A.M.) screened the majority of all participants for study inclusion, whereas a variety of trained team members consented participants into the study. All data will be openly available in FITBIR at fitbir.nih.gov (reference number FITBIR-STUDY0000339) at the conclusion of the study.

## Clinical assessments

A Common Data Elements battery of tests was administered at both visits (Supplemental Methods; Supplemental Table 1). Measures included medical history, a semi-structured interview to assess history of TBI (The New Mexico Assessment of Pediatric Traumatic Brain Injury)^25^, the Alcohol, Smoking and Substance Involvement Screening Test (ASSIST), self- and parent reports of concussion symptom severity for both retrospective and current visits (Post-Concussion Symptom Inventory [PCSI]), Patient Reported Outcomes Measurement Information System (PROMIS) for sleep, anxiety, and depression, a brief pain rating (0–10 Likert scale), self-report of Tanner stage of development, Headache Impact Test (HIT-6), the Strengths and Difficulties Questionnaire (SDQ), Conflict and Behavioral Questionnaire (CBQ), Pediatric Quality of Life Inventory (PedsQL – Generic Core), and the Glasgow Outcome Scale Extended (GOS-E) Pediatric Revision. Parental distress was measured with the Brief Symptom Inventory (BSI-18). The PCSI, CBQ, and PedsQL served as primary clinical outcome measures. Recovery from post-concussive symptoms (i.e., symptomatic versus asymptomatic) was determined using a normative rather than simple change approach to reduce false positives among HC^8^.

The cognitive battery included tests of premorbid cognitive ability (Wide Range Achievement Test [WRAT4]), a shortened measure of effort (Test of Memory Malingering [TOMMe10]), and selected tests from the Delis-Kaplan Executive Function System (D-KEFS), the Hopkins Verbal Learning Test-Revised (HVLT-R), and Wechsler Intelligence Scales depending on initial age at assessment. Composite measures of attention (DKEFS color-word interference conditions 1–3), processing speed (WAIS-IV/WISC-V digit symbol coding and symbol search), working memory (WISC-V/WAIS-IV digit span backwards trial), executive function (DKEFS trail making test condition 4, verbal fluency, color-word interference condition 4) and long-term memory recall (HVLT-R Delay) were compiled to create specific cognitive domains by averaging t-scores from individual tests. Attention and processing speed served as the primary cognitive outcome measures.

## Blood-based biomarker analyses

Whole blood samples were obtained at each visit using standard venipuncture procedures, centrifuged for 15 min at 1500 relative centrifugal force, aliquoted into 400–500 µl cryovials, and stored in a −80 °C freezer. Frozen plasma samples were shipped overnight on dry ice and processed blinded to group affiliation using the Simoa^®^ Quanterix™ HD-X platform. Assays included UCH-L1, NFL, GFAP, total tau (run simultaneously on the N4PA assay) and pTau181, and were run across two separate batches. Each batch was run with appropriate standards and controls to ensure reliability, and batch was used as a nuisance factor for all analyses. Longitudinal samples from the same individual were always run on the same plate. There were no samples with concentrations below minimum levels of detection across all assays, but several samples were below levels of quantification for UCH-L (see Supplemental Table 2). A much higher percentage of UCH-L1 samples had coefficients of variation (COV) above 30% (40.4% affected) relative to other biomarkers. Analyses were repeated with and without samples with COV above 30% for UCH-L1, GFAP and pTau181. However, results remained statistically similar with and without high COV samples. Different versions of the pTau181 kit were utilized to process samples and protein concentration levels were transformed per the manufacturer’s recommendations (see Supplemental Table 2). Finally, all concentration values were log-transformed to improve distributional properties.

## Analytic plan

All analyses were conducted in SPSS (IBM Corp. Version 20.0). Generalized estimating equations (GEE) or Generalized Linear Models (GLM) were used for demographic, clinical and blood-based biomarker comparisons, with distribution (negative binomial, normal or gamma) selected based on information criterion results and visual inspection. Results within each domain were adjusted for multiple comparisons using the Bonferroni correction. GLM examined the relationship between clinical indicators of injury severity (PCS, PTA/LOC, etc.) and protein concentration levels. Individuals who reported isolated LOC, isolated PTA or both LOC and PTA were combined into a single group given that these medical concepts are frequently conflated during injury reporting from a lay perspective^26–29^. Intraclass correlation coefficients^30^ were used to examine test-retest reliability for HC blood samples [ICC(2,1)]. Reliability estimates were categorized as *poor* (≤ 0.39), *fair* (0.40–0.59), *good* (0.60–0.74) or *excellent*(≥ 0.75) based on published guidelines^31^. Effect sizes were categorized based on the criteria put forth by Cohen for both correlation statistics and between group differences (small [|r|=0.1–0.3 or |d|=0.2–0.5]; medium [|r|=0.31–0.5 or |d|=0.51–0.8] or large [|r|>0.51 or |d|>0.8]).

Finally, Random forests were used to classify pmTBI from HC, and to determine sensitivity of these biomarkers to PTA/LOC. Random forest is a supervised ensemble learning algorithm^32,33^ in which multiple classification trees (a “forest”) are fit on bootstrapped samples. Each tree partitions the data into a random subset of predictor variables to optimally separate diagnostic categories. Random forest does not have distributional model assumptions, and automatically employs external cross-validation by predicting group membership based on trees estimated from subsamples. The bootstrap aggregating technique minimizes data overfitting. Random forest analyses were performed in R software (4.3.1) using package “randomForestSRC” (3.2.2) function “rfsrc” with 1000 trees.

## Results

### Sample demographics

**Fig. 1:**
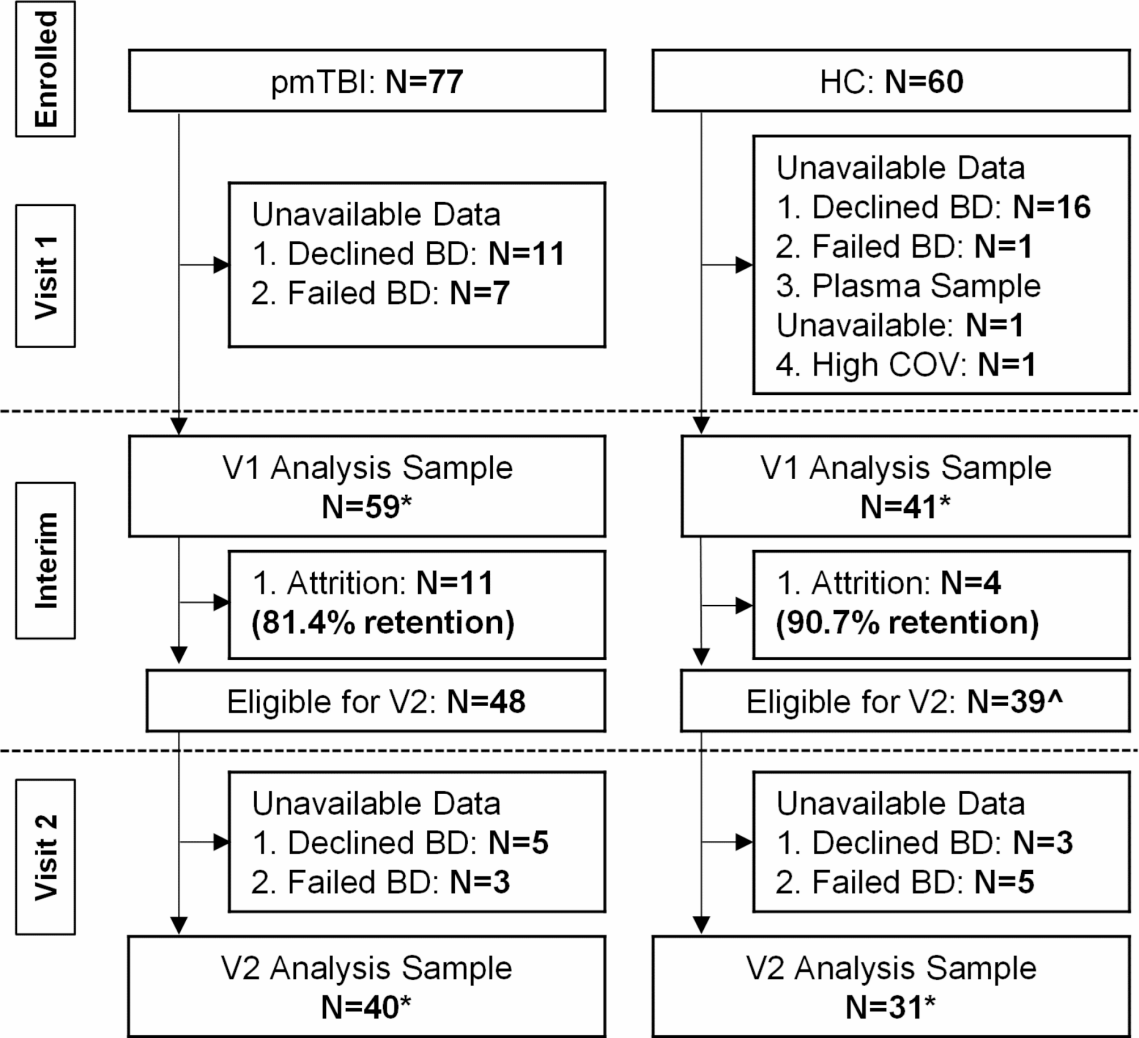
Participant Flowchart and CONSORT diagram. Flowchart of participant enrollment, inclusion, and data quality assurance from the first visit (V1; approximately 7 days post-injury) and second visit (V2; approximately 4 months post-injury). Blood draws (BD) were obtained from both patients with pediatric mild traumatic brain injury (pmTBI) as well as healthy controls (HC). The interim period refers to the time between study visits when attrition may have occurred. Asterisks (*) denote the number of samples with available blood biomarker data used in final analyses at each visit. The biomarker sample sizes slightly varied relative to clinical data sample sizes due to issues with blood collection or quality assurance. The caret (^) symbol indicates that blood-based biomarker data from two eligible HC was successfully collected at V2 but not V1.

Whole blood samples were obtained from 59 pmTBI (28 females; age 14.9 ± 2.7). V1 occurred 7.1 ± 2.2 days post-injury (range 0–10 days), with V2 occurring approximately four months later (133.3 ± 15.3 days post-injury). Blood-based biomarkers were analyzed from 41 HC (20 females; age 14.3 ± 2.8) evaluated at similar intervals (135.5 ± 18.9 days between visits). Twenty-seven additional participants (11 pmTBI and 16 HC) declined the blood draw, eight attempts (7 pmTBI, 1 HC) at blood collection blood were unsuccessful, and plasma was unable to be collected from one HC (see Fig. 1 and Supplemental materials for full details). A total of 48 pmTBI and 39 HC returned for V2. Eight participants (5 pmTBI, 3 HC) declined the blood draw during V2 and eight (3 pmTBI, 5 HC) blood collection attempts were unsuccessful. Blood samples were therefore available from 40 pmTBI (17 females) and 31 HC (14 females) for V2.

**Fig. 2:**
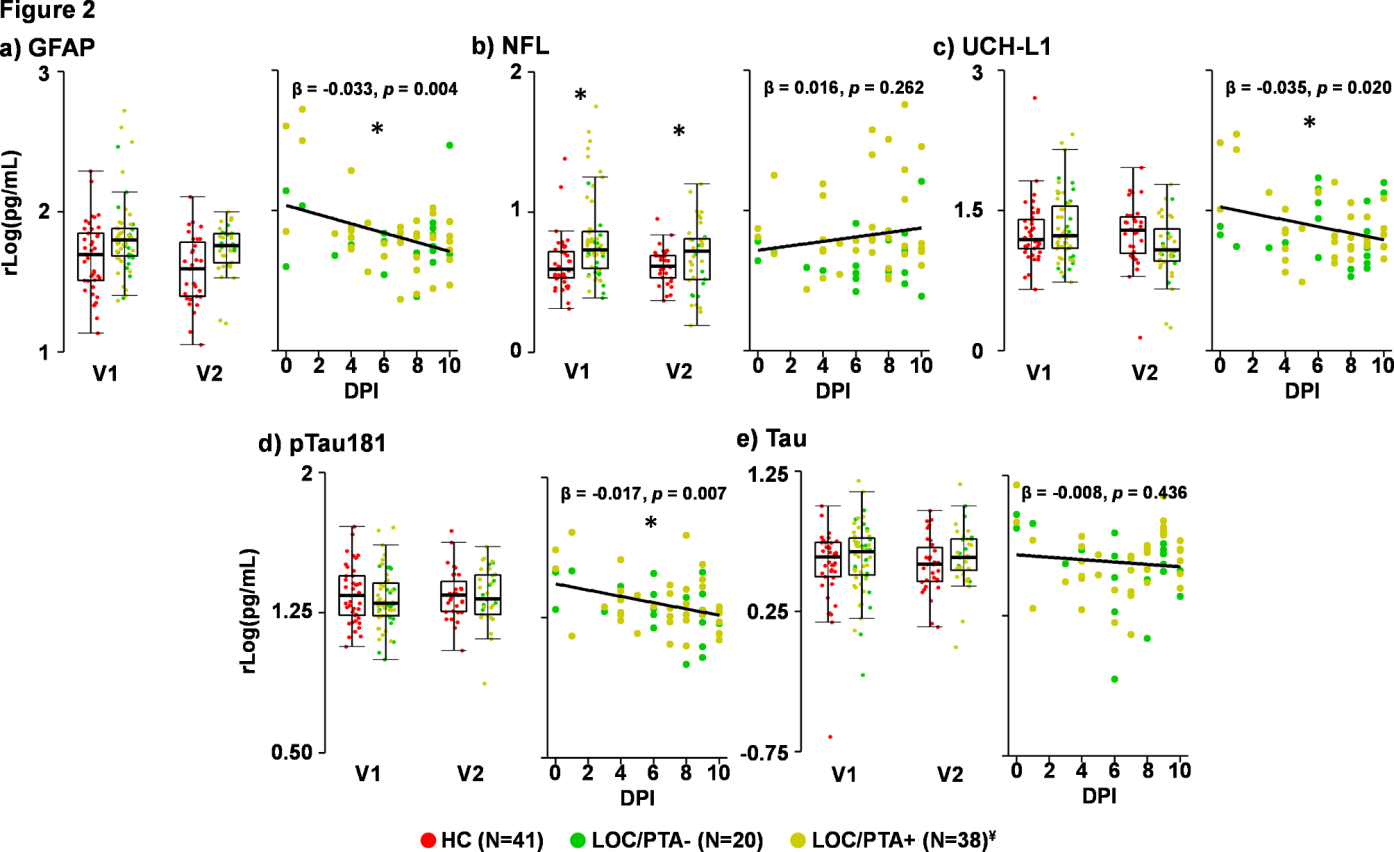
Blood-based biomarker results. Box-and-scatter plots presenting glial fibrillary acidic protein (GFAP; Panel **a**), neurofilament light chain (NFL; Panel **b**), ubiquitin C-terminal hydrolase L1 (UCH-L1; Panel **c**), phosphorylated tau 181 (pTau181; Panel **d**) and total tau (Panel **e**) concentration levels (unit = log of picogram/milliliter [pg/mL] plus constant) for patients with pediatric mild traumatic brain injury (pmTBI; yellow and green dots) as well as healthy controls (HC; red dots). Data are presented separately for the first (V1; approximately 7 days post-injury) and second (V2; approximately 4 months post-injury) visits, and are residualized (rLog) for batch effects. Asterisks denote significant group differences or significant correlations with days post-injury. Patient data are further stratified by presence (yellow) or absence (green) of post-traumatic amnesia and/or loss of consciousness (PTA/LOC). Separate regression plots (β = unstandardized beta) are presented depicting associations with days post-injury (DPI) for the patient group only. The frequency pmTBI enrolled on each day post-injury is also presented in this plot. Sample sizes are included in the figure, and ^¥^ denotes that there was a single pmTBI who was missing PTA/LOC status at V1.

Demographics and injury characteristic data are presented in Table 1, whereas Table 2 presents central tendency data for all clinical and neuropsychological results. Groups did not differ on biological sex, age, self-reported Tanner stage of development or handedness (all *p*’s ≥ 0.05). Significant group differences existed for previous number of head injuries (χ^2^ = 6.53, *p* = 0.011; pmTBI = 18.6%, HC = 2.3%), parental self-reported psychopathology (BSI-18 *Wald-χ*^*2*^ = 8.88; *p* = 0.003; pmTBI > HC), premorbid reading ability (WRAT-IV *Wald-χ*^*2*^ = 21.70; *p* ≤ 0.001; pmTBI < HC) and effort (TOMMe10 *Wald-χ*^*2*^ = 12.06; *p =* 0.001; pmTBI < HC). Reading ability and effort were therefore used as additional covariates during neuropsychological analyses. A total of two pmTBI (6.06%) had positive CT scans indicative of small areas of hemorrhage, and both of these pmTBI also self-reported positive PTA/LOC. No participants experienced an additional concussion between V1 and V2 as confirmed by our semi-structured interview. The frequency of pmTBI enrolled at each day post-injury is depicted in the scatter plots in Fig. 2. See Table 1 for details on the mechanism of injury, which were determined using published methods^34,35^.

**Table 1:** Demographics and injury characteristic data.

	V1 pmTBI (*N* = 59)	V1 HC (*N* = 43)
Age	15.5(13.42–17.08)	14.42(12.58–16.17)
Sex (% Female)	47.46%	46.51%
Tanner Stage of Development	4(3–4)	4(3–4)
Handedness (% Right)	91.5%	93.0%
Parent BSI-18^a^	4(1–7.5)	2(1–6)
pmTBI Hx^a^	18.97%	2.33%
Injury Characteristics		
Loss of Consciousness	44.83%	-
Post-Traumatic Amnesia	37.93%	-
Positive CT Scan	2/33	-
Mechanism of Injury		
Struck by Object	10.34%	-
Struck by Person	15.52%	-
Fall	29.31%	-
MVC	43.1%	-
Assault	0.00%	-
Bicycle	0.00%	-
Other	1.72%	-
Sport or Recreation Related	51.72%	-

*Notes*: V1 = Visit 1 (~ 7 days post-injury); HC = healthy control; pmTBI = pediatric mild traumatic brain injury; BSI = Brief Symptom Inventory-18; MVC = motor vehicle crash; Hx = history. Data are either formatted at mean ± standard deviation or median (interquartile range) based on distribution properties. ^a^ = Group main effect. Clinical data are included from all participants from whom blood samples were obtained.

**Table 2:** Clinical and neuropsychological data.

Metric	Outcome	V1 pmTBI (*N* = 59)	V1 HC (*N* = 43)	V2 pmTBI (*N* = 48)	V2 HC (*N* = 39)
Symptom measures					
PCSI (% Max)^†^	P	17.5(5.9–37.3)	3.1(0.8–7.9)	5.6(2.4–21.8)	4.8(1.2–10.6)
PROMIS Sleep^*^	S	19.0 ± 6.7	14.2 ± 4.7	18.1 ± 6.3	15.4 ± 4.9
PROMIS Anxiety^*^	S	4(0–11)	0.5(0–4)	2(0–10.5)	4(0–5)
PROMIS Depression	S	6(1–12)	1.5(0–4)	1(0–9)	3(0–6.5)
Pain Scale^*^	S	4(1–6)	0(0–1)	1(0–3)	0(0–1)
HIT-6^†^	S	52.6 ± 10.8	41.0 ± 5.6	48.5 ± 8.3	44.4 ± 7.7
Behavioral & outcome measures					
CBQ	P	2(0–3)	1(0–2)	1(0–2)	1(0–2)
PedsQL	P	N/A	N/A	82.7 ± 10.7	86.5 ± 8.3
SDQ	S	N/A	N/A	7.4 ± 4.4	4.3 ± 3.3
GOS-E^†^	S	1(1–3)	1(1–1)	1(1–1)	1(1–1)
Cognitive measures					
TOMMe10^*^	S	10(9–10)	10(10–10)	10(9–10)	10(10–10)
WRAT-IV^*^	S	48.1 ± 9.8	57.7 ± 11.3	51.0 ± 8.5	61.1 ± 11.3
PS	P	45.0 ± 8.3	50.3 ± 8.9	50.6 ± 7.7	53.5 ± 8.8
AT^†^	P	46.9 ± 9.7	52.9 ± 6.2	51.0 ± 7.7	53.9 ± 4.9
WM^*^	S	45.9 ± 7.6	53.1 ± 8.1	46.4 ± 10.2	55.0 ± 11.9
EF^*^	S	45.8 ± 7.6	51.4 ± 6.2	50.1 ± 7.2	54.0 ± 6.0
HVLT-R Delay^*^	S	7.1 ± 2.3	9.0 ± 2.0	7.2 ± 2.3	8.7 ± 2.3

*Notes*: V1 = Visit 1 (~ 7 days post-injury); V2 = Visit 2 (~ 4 months post-injury); HC = healthy control; pmTBI = pediatric mild traumatic brain injury; N/A = Not applicable; PCSI = Post-Concussion Symptom Inventory (presented as percent of maximum score to account for age-related scale differences); PROMIS = Patient Reported Outcomes Measurement Information System; HIT-6 = Headache Impact Test; CBQ = Conflict Behavior Questionnaire; SDQ = Strengths and Difficulties Questionnaire; PedsQL = Pediatric Quality of Life Inventory; GOS-E = Glasgow Outcome Scale Extended; TOMMe10 = Test of Memory Malingering – 10-item short version; WRAT-IV = Wide Range Achievement Test 4; PS = processing speed; AT = attention; WM = working memory; EF = executive function; HVLT-R Delay = Delayed recall on Hopkins Verbal Learning Test-Revised (measure of long-term memory). Data are either formatted at mean ± standard deviation or median (interquartile range).*=Group main effect and †= Group × Visit interaction. Clinical data are included from all participants from whom blood samples were obtained.

## Clinical outcomes

A significant Group×Visit interaction was observed for self-reported PCS severity (PCSI *Wald-χ*^*2*^ = 16.04 *p* < 0.001), with increased PCSI scores (pmTBI > HC) at V1 (*Wald-χ*^*2*^ = 74.62; *p* < 0.001; 49.12% symptomatic) relative to V2 (*Wald-χ*^*2*^ = 8.38; *p* = 0.004; 14.89% symptomatic). The primary quality of life (PedsQL) and self-reported behavioral disturbance (CBQ) measures were not significantly different between groups (all *p*’s > 0.05).

Analyses of secondary clinical measurements also indicated a significant Group×Visit interaction for headaches (HIT-6 *Wald-χ*^*2*^ = 13.24; *p* < 0.001 ≤ Bonferroni corrected 0.05/7 = 0.007), with pmTBI demonstrating greater symptom burden at V1 (*p* < 0.001) but not at V2 (*p* = 0.151). There was also a Group×Visit interaction for the Glasgow Outcome Scale Extended (GOS-E *Wald-χ*^*2*^ = 8.71, *p =* 0.003), with pmTBI exhibiting significantly greater scores (indicating worse outcome) at V1 (*Wald-χ*^*2*^ = 27.98; *p* < 0.001) relative to V2 (*Wald-χ*^*2*^ = 4.03; *p* = 0.045). Main effects of Group (pmTBI > HC) were observed for pain (Pain Scale *Wald-χ*^*2*^ = 20.31; *p* < 0.001), sleep (PROMIS Sleep *Wald-χ*^*2*^ = 11.67; *p =* 0.001) and anxiety (PROMIS Anxiety *Wald-χ*^*2*^ = 11.31; *p =* 0.001), as well as a main effect of Visit (V2 > V1) for anxiety (*Wald-χ*^*2*^ = 10.10; *p =* 0.001). There were no significant effects for the PROMIS depression scale following Bonferroni correction.

For primary cognitive domains, a significant main effect of Visit (V2 > V1) was observed for processing speed (*Wald-χ*^*2*^ *=* 27.89; *p <* 0.001), whereas a significant Group×Visit interaction was found for attention (*Wald-χ*^*2*^ = 6.86; *p* = 0.009; Bonferroni-corrected *p* < 0.025). However, follow-up tests revealed that group differences in attention were not statistically significant at either V1 (*p* = 0.085) or V2 (*p* = 0.807). Significant main effects of Group (HC > pmTBI) were observed (Bonferroni-corrected *p* < 0.0167) for secondary cognitive domains of executive function (Wald-*χ*^2^ = 5.84; *p* = 0.016), delayed recall (*Wald-χ*^*2*^ = 9.68; *p* = 0.002) and working memory (*Wald-χ*^*2*^ *=* 5.93; *p* = 0.015).

### Blood-based Biomarker results

Investigations on test-retest reliability for blood biomarkers were limited to HC samples (*N* = 30) given a priori predictions of changing values following pmTBI. Reliability estimates were in the fair range for NFL (ICC = 0.57), good range for UCH-L1 (ICC = 0.73) and total Tau (ICC = 0.69), and excellent range for GFAP (ICC = 0.90) and pTau181 (ICC = 0.84) across the four-month assessment window for HC.

A series of 2 × 2 (Group×Visit) GEE models evaluated potential differences in protein concentrations with batch as a covariate (Fig. 2). No significant effects were associated with total tau or pTau181 (all *p’*s > 0.05). NFL exhibited significant main effects for both Group (*Wald-χ*^*2*^ = 15.50; *p <* 0.001; pmTBI > HC) and Visit (*Wald-χ*^*2*^ = 4.27; *p =* 0.039; V1 > V2). Medium effect sizes were present at V1 for NFL (*p <* 0.001; Cohen’s d = 0.72), with evidence of continued elevated protein levels and small effect sizes at V2 (*p =* 0.015; Cohen’s d = 0.41). Both UCH-L1 (*Wald-χ*^*2*^ = 4.35; *p =* 0.037) and GFAP (*Wald-χ*^*2*^ = 6.42 *p =* 0.011) exhibited significant main effects of Visit, most likely a result of higher values in the pmTBI group at V1 (UCH-L1 V1 Cohen’s d = 0.30; GFAP V1 Cohen’s d = 0.24) in conjunction with limited statistical power.

Secondary GLM examined the relationship between protein concentration levels and days post-injury within the pmTBI group only (Fig. 2). Significant negative correlations for GFAP (*r*=−0.37; *p* = 0.004), UCH-L1 (*r*=−0.30; *p* = 0.020) and pTau181 (*r*=−0.35; *p* = 0.007) existed with days post-injury, with all of the correlations being in the small to medium effect size range. In contrast, total tau and NFL were not associated with days post-injury (both *p*’s > 0.05).

### Relationships between blood-based biomarkers and clinical findings

The next series of analyses examined the potential sensitivity of biomarkers to the presence (yellow dots; *N* = 38) or absence (green dots; *N* = 20) of PTA/LOC (Fig. 2), concussion history, and PCS at V1 in pmTBI patients only. Data on presence/absence of PTA/LOC and concussion history was missing for a single pmTBI at V1. Results indicated that pmTBI with PTA/LOC + had significantly higher NFL levels at V1 relative to PTA/LOC- with medium effect sizes (*Wald-χ*^*2*^ = 6.10; *p =* 0.014; Cohen’s d = 0.77). In contrast, there were no significant effects of PTA/LOC for UCH-L1, GFAP, total tau, or pTau181. Neither concussion history nor PCSI scores were significantly associated with any blood-based biomarker concentration levels (all *p’s* > 0.05). However, similar to previous findings with days post-injury, there was qualitative evidence for both GFAP and UCH-L1 being increased in pmTBI with PTA/LOC + up to three days post-injury (see proportion of yellow and green points at early days post-injury in Fig. 2).

**Fig. 3:**
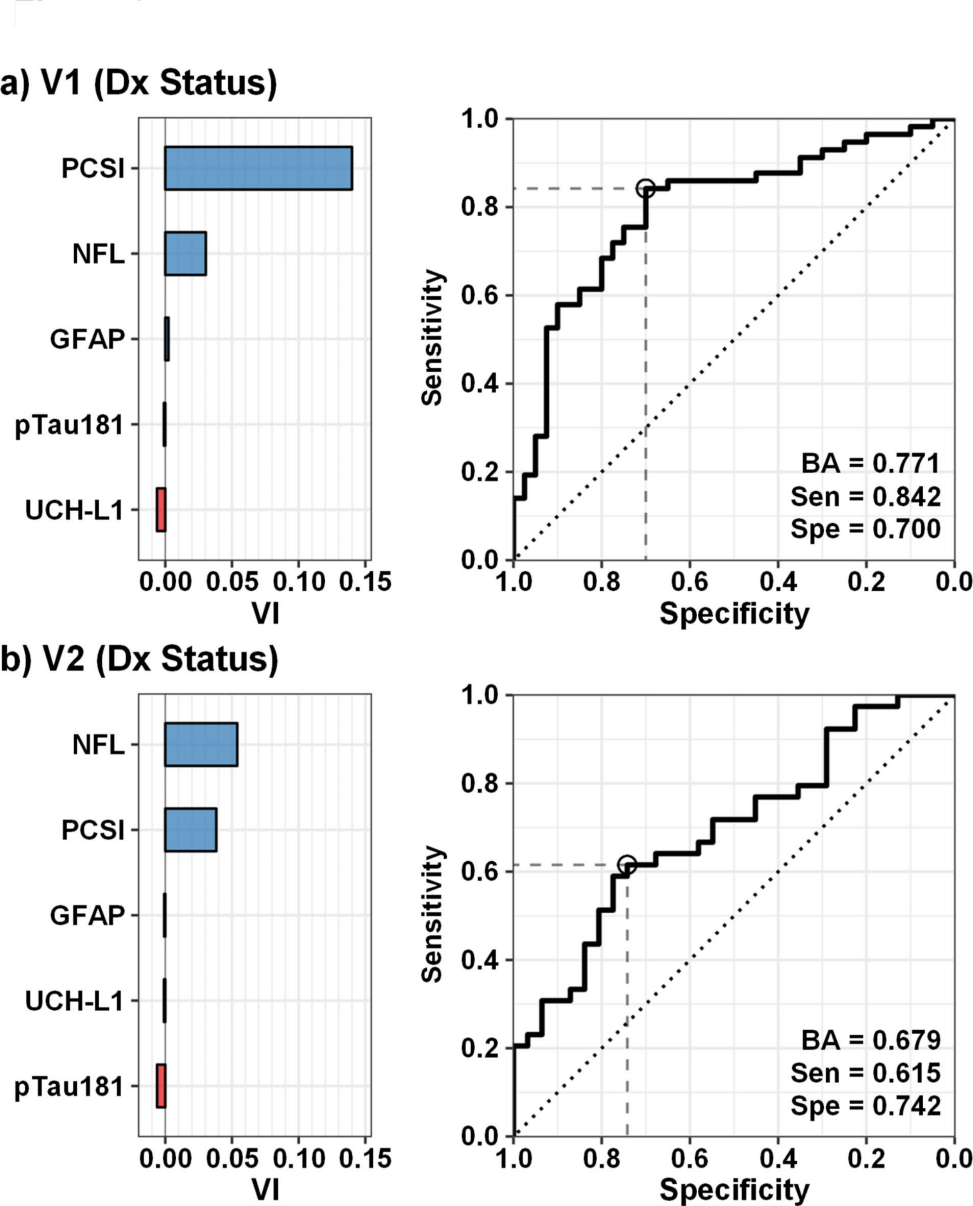
Group classification using Random forest machine learning algorithm. Receiver operating characteristics and variable importance (VI) results from the Random forest supervised machine learning algorithm for classifying diagnostic (Dx) status in patients with pediatric mild traumatic brain injury (pmTBI) relative to healthy controls (HC). The model included Post-concussion Symptom Inventory percent total score [PCSI], glial fibrillary acidic protein (GFAP), neurofilament light chain (NFL), ubiquitin C-terminal hydrolase L1 (UCH-L1) and phosphorylated tau 181 (pTau181). Results are presented separately for the first (V1; Panel a) and second (V2; Panel b) visits and include balanced accuracy (BA), specificity (Spe), and sensitivity (Sen). The intersection of optimal sensitivity/specificity is denoted with a circle. NFL contributed more towards diagnostic accuracy relative to PCSI scores at V2.

**Fig. 4:**
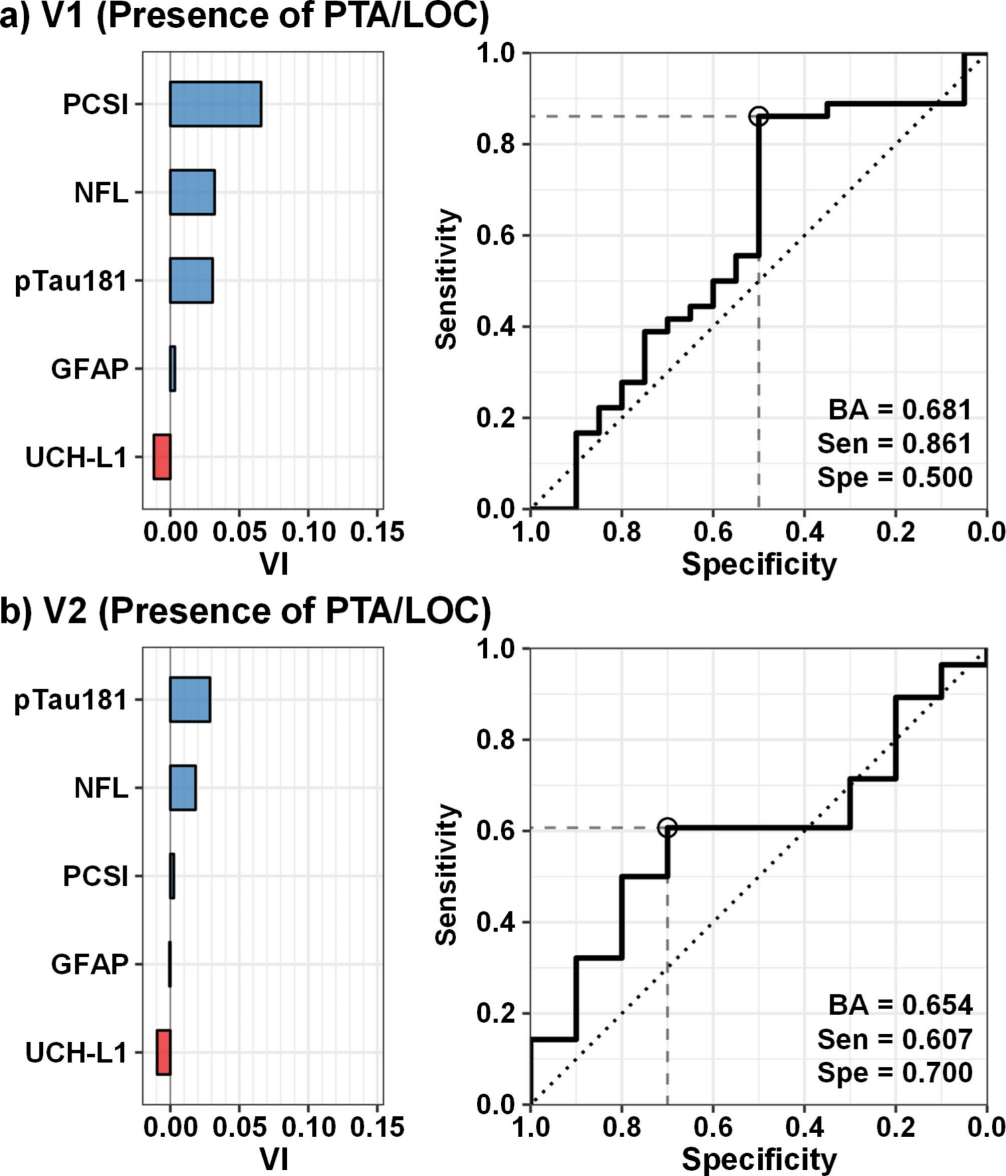
Classification of injury severity using Random forest machine learning algorithm. Receiver operating characteristics and variable importance (VI) results from the Random forest supervised machine learning algorithm for classifying patients with pediatric mild traumatic brain injury based on the presence or absence of post-traumatic amnesia and/or loss of consciousness (PTA/LOC). The model included Post-Concussion Symptom Inventory percent total score [PCSI], glial fibrillary acidic protein (GFAP), neurofilament light chain (NFL), ubiquitin C-terminal hydrolase L1 (UCH-L1) and phosphorylated tau 181 (pTau181). Results are presented separately for the first (V1; Panel a) and second (V2; Panel b) visits, and include balanced accuracy (BA), specificity (Spe), and sensitivity (Sen). The intersection of optimal sensitivity/specificity is denoted with a circle. Among the biomarkers that were examined, NFL showed the greatest promise for detecting PTA/LOC at V1, although sensitivity was higher than specificity.

Finally, a supervised machine learning algorithm (Random forests) examined the classification accuracy of PCSI and blood-based biomarkers separately for both diagnosis (pmTBI and HC) and presence/absence of PTA/LOC (pmTBI only) separately for V1 and V2. Results indicated that balanced accuracy for diagnosis was highest at V1 (Fig. 3a; BA = 0.77), with greater sensitivity (0.84) than specificity (0.70). PCSI, NFL, and GFAP respectively exhibited the highest variable importance scores. As expected, the balanced accuracy of the model decreased at V2 (Fig. 3b; BA = 0.68). However, NFL exhibited a relatively higher variable importance score than PCSI. Similarly, both NFL and pTau181 variable importance scores were higher or equivalent relative to the PCSI for classifying the presence or absence of PTA/LOC in pmTBI at both V1 (Fig. 4a) and V2 (Fig. 4b), with modest balanced accuracies for both models (V1 = 0.68; V2 = 0.65).

## Discussion

Various expert consensus panels recommend reliance on self-reported symptoms, PTA, and LOC to diagnose injury severity post-pmTBI, with self-reported symptoms primarily used to determine injury recovery and inform return-to-play decisions^5,36^. Blood-based biomarkers have been proposed to represent a surrogate marker for capturing temporally dynamic patterns of injury observed in preclinical models, but applications to pmTBI samples have been limited^18,21^. Importantly, a recent large N reference study^20^ demonstrated that protein concentration levels change during neurodevelopment, highlighting the need to specifically examine pediatric injury. Current results preliminarily suggest a relatively short diagnostic window for plasma GFAP, UCH-L1 and pTau181 (< 72 h) concentration levels in children, with NFL exhibiting statistically significant sensitivity and specificity up to four months post-pmTBI.

Multidimensional post-concussive symptoms (cognitive, physical and emotional) existed at the sub-acute visit, which resolved for some, but not all domains at four months post-injury. Similarly, both current and prior findings suggest that subtle cognitive deficits were present on neuropsychological testing during the first few weeks post-injury^37–39^, with some persisting for 4 months post-injury for executive dysfunction, working memory and long-term memory. A sex- and age-matched control group underwent identical procedures at equivalent time points to control for multiple potential confounders in clinical and cognitive testing (e.g., practice effects, typical neurodevelopment across the repeat visits). The healthy control group data were also used to examine the test-retest reliabilities of blood-based biomarkers across a four-month visit window. Results suggested that test-retest reliabilities were in the good range for the majority of tested biomarkers (GFAP, total tau, pTau181 and UCH-L1) with the exception of NFL (fair range). A high percentage of UCH-L1 samples exhibited coefficients of variation above 0.30. Similar results for this protein have been previously observed with Quanterix’s multiplex assay in other TBI samples^40^.

Current findings indicated no significant differences between pmTBI and HC for either plasma UCH-L1 or GFAP levels at approximately 7 days post-injury. Other cross-sectional studies have suggested that both GFAP^41^ and UCH-L1^42^ levels are higher in pmTBI with positive CT scans, as well as elevated in non-lesioned pmTBI relative to controls^43–45^. However, the majority of these studies were predominantly limited to acute injury stages (0–72 h)^41–44^. Current results similarly indicated a significant negative relationship between both markers and days post-injury in the sub-acute injury phase, suggesting that more rapid sampling time (e.g., within 48 h) is a critical factor for detecting changes in GFAP and UCH-L1 concentrations levels. In contrast, NFL was elevated at approximately 7 days post-injury and remained statistically elevated up to 4 months post-pmTBI even though it exhibited the smallest test-retest reliability coefficient. Previous studies have indicated that NFL exhibits a dose-dependent response curve for mild, moderate and severe TBI during acute and more chronic injury stages^46 ^and is also associated with recorded sub-concussive blows in boxers^16^. Neurofilaments are more abundant in large caliber, myelinated axons relative to dendrites and the soma, and elevations in the blood stream are therefore posited to be reflective of axonal pathololgy^47^. Other adult mTBI studies have also reported elevated NFL up to one month post-injury^16,19^, as well as increasing NFL levels in more severely injured athletes even at return to play^14^. In contrast, negative findings of long term NFL elevations were observed in a previous pmTBI study^22^. Differences between current and previous findings^22^ may have resulted from a lack of power at long-term follow-up in the previous study (*N* = 18), differences in injury mechanisms and severity (Emergency room sample versus sports-related concussion), differences in sample acquisition (4 months versus 6 month follow-up), or a combination of these factors. Collectively, these findings suggest ongoing physiological processes associated with NFL protein release that may occur in the weeks to months following mTBI.

pTau181 also demonstrated a significant negative relationship with days-post injury but did not significantly differ between groups. In contrast, total tau was neither significantly different between groups nor associated with days post-injury. Previous pmTBI studies have suggested that total tau stratifies patients based on Glasgow Coma Scale score^48 ^and symptom severity^22^, but not for positive CT scans^48^. More recent work indicates minimal correspondence between serum levels of total tau and tau derived from cerebral spinal fluid^49^, questioning the specificity of the Quanterix total tau blood-based marker for head injury.

Mild TBI exists on a spectrum^5^, and the Bradford-Hill criteria suggests that biomarkers should exhibit a dose-dependent relationship with injury severity and be predictive of outcomes. Adult mTBI studies indicate that GFAP classifies patients with and without CT lesions^13^ and those with and without PTA/LOC^14,15^. Although these GFAP findings were not replicated in the current pediatric sample, NFL exhibited both significant group differences and modest classification accuracy for stratification of pmTBI based on the presence of PTA/LOC similar to larger adult studies in sports-related concussion^14^. Importantly, medical nuances regarding alterations in mental status are frequently conflated by lay populations^26–29^. As a result, the current study combined individuals who reported PTA, LOC or both injury characteristics into a single group for our primary analyses and in an attempt to replicate previous findings from the adult literature^14^.

Acute GFAP has also been shown to predict symptom burden at 24 h at one month post-injury^50 ^and risk for vomiting^45 ^following pmTBI, but these findings were not replicated in another study examining the prognostic utility of acute GFAP and UCH-L1 with outcomes at one month^51^. The current study also did not observe any associations between blood-based biomarkers and post-concussive symptom load, with NFL outperforming PCSI in diagnostic classification at V2 (Fig. 3b). PCS are non-specific, represent diverse (e.g., cognitive, somatic, emotional and neurosensory) complaints, exhibit relatively poor psychometric properties, and symptom resolution is influenced by age and other sample characteristics^8,52^. Although the debate about how PCS versus alterations in mental status influence mTBI diagnosis is highly relevant from a clinical standpoint^36^, detecting relationships between biomarkers and PCS in simple linear models will likely be more challenging.

Previous research has suggested that different blood-based biomarker index a unique pathophysiological construct at the cellular level (e.g., UCH-L1 = neuronal cell body injury; NFL/tau = axonal injury; GFAP = astrocytic gliosis)^11,12^. However, emerging preclinical and clinical evidence suggests that individual blood-based biomarkers are associated with multiple different types of histopathology, are elevated following general trauma in the absence of neurotrauma, are elevated following prolonged anesthetic exposure, and may increase as a function of age and renal clearance^53–58^. Similarly, a recent large imaging study (*N*= 2869) suggested that blood-based biomarker levels (GFAP, total tau, UCH-L1 and NFL) were more indicative of injury severity and total intracranial injury burden rather than representing specific pathoanatomical injury sub-types based on imaging^59^. Collectively, these data suggest that multiple biological processes (e.g., change in blood-brain barrier permeability, renal clearance, hypoxia) contribute to changes in individual protein concentrations in blood. Thus, while a growing body of literature suggests clear diagnostic and some prognostic indications for blood biomarkers^11,12^, additional research is required to determine suitability for theragnostic or target engagement purposes.

There are several limitations to the study. Foremost, pmTBI were assessed over a 10-day range during the first visit, which may be too protracted to capture the more rapid resolution of several protein concentration levels in the blood stream (GFAP, UCH-L1 and pTau181). Future pediatric studies should consider blood-based biomarker assessments within 48 h as well as more long-term assessments to investigate the full range of diagnostic and prognostic sensitivity. Second, the overall sample size was modest, which may limit the generalizability of our findings. Similarly, the current study was unable to examine relationships between blood-based results and structural findings on traditional imaging due to low (i.e., only 2 patients with positive CT findings) statistical power^41,42^. Third, the selected biomarker panel was primarily focused on cellular rather than vascular or inflammatory markers of injury. There are limited studies examining blood biomarkers of vascular injury in either adult or pediatric mTBI^60,61 ^and a single, small study suggesting that inflammatory markers are prognostic following pmTBI^62^. Fourth, the study did not assess more ecologically valid outcome measures such as time to return to school or play post-injury.

In summary, fluid biomarkers may provide a cost-effective, rapid screener for pmTBI that can be deployed across diverse point-of-care settings (sidelines to generalist offices to emergency room). This may eventually reduce the use of CT scans that have low sensitivity to diffuse injuries and confer radiation risk in a vulnerable population^7,18^. Our initial findings highlight dynamic fluctuations between blood-based biomarkers and time post-injury, with evidence that changes in NFL may persist up to four months post-injury. Collectively current and previous results^14 ^highlight the importance of different biomarkers at acute (e.g., GFAP and UCH-L1) versus more sub-acute/early chronic injury stages (NFL). These results parallel emerging evidence from advanced imaging^63^, and suggest delayed and ongoing pathophysiological processes that continue for months following pmTBI.

## Electronic supplementary material

Below is the link to the electronic supplementary material.

Supplementary Material 1

## Data Availability

The datasets generated during and analyzed during the current study will be available in the FITBIR repository: fitbir.nih.gov, reference number FITBIR-STUDY0000339 (https://fitbir.nih.gov/portal/study/viewStudyAction!view.action?studyId=FITBIR-STUDY0000339). The datasets generated during and analyzed during the current study are currently available from the corresponding author on reasonable request.
