# Supplementary material for: Blood-based biomarkers suggest prolonged axonal Injury following pediatric mild traumatic brain injury
Source: Sci Rep. 2025 Feb 4;15:4189. doi: 10.1038/s41598-024-84053-4 (PMC11794578; doi:10.1038/s41598-024-84053-4)
Supplement: Supplementary file 1 — Supplementary Material 1 [file 41598_2024_84053_MOESM1_ESM.docx]

**Supplemental Materials**

*Participants*

A total of 77 pediatric mild traumatic brain injury patients (pmTBI) were consecutively recruited from two local Emergency Room and Urgent Care departments along with 60 statistically matched (sex and age) healthy controls (HC) to participate in the study. A total of 27 participants (11 pmTBI and 16 HC) declined the blood draw, eight (7 pmTBI and 1 HC) attempts at collecting blood were unsuccessful (i.e., missed vein), one HC did not have plasma collected at the first visit (V1) and one HC was eliminated for having multiple samples with high coefficients of variation (COV). The final sample with useable blood samples included 59 pmTBI (28 females; age 14.9±2.7; 7.1±2.2 days post-injury) and 41 HC (20 females; age 14.3±2.8) for the V1 visit. All participants who were eliminated for having multiple samples with high COV or who had no plasma collected at V1 were considered to be eligible for V2 analysis.

From this subset, study attrition occurred for 11 pmTBI (81.4% retention without study excludes) and 4 HC (90.7% retention). This resulted in 48 pmTBI and 39 HC eligible for a blood draw during their second visit (V2). Eight participants (5 pmTBI and 3HC) declined the blood draw at V2 but completed clinical testing. Blood collection attempts in an additional eight (3 pmTBI and 5 HC) were unsuccessful. This led to a total of 40 pmTBI (17 females; 133.3±15.3 days post-injury; 126.6±14.8 days between visits;) and 31 HC (14 females; 135.5±18.9 days between visits) with V2 biomarker data.

*Common Data Element Measures*

A battery of clinical and neuropsychological measures was administered to pmTBI and HC at both V1 and V2. Measures included medical history, the New Mexico Assessment of Pediatric Traumatic Brain Injury semi-structured interview ^1^, the Alcohol, Smoking and Substance Involvement Screening Test (ASSIST) ^2^, self- and parent reports of concussion symptom severity for both retrospective and current periods (Post-Concussion Symptom Inventory [PCSI]) ^3,4^, Patient Reported Outcomes Measurement Information System (PROMIS) for sleep ^5^,anxiety, and depression ^6^, a brief pain rating (0-10 Likert scale) ^7^, self-report of Tanner stage of development ^8^, Headache Impact Test (HIT-6) ^9^, the Strengths and Difficulties Questionnaire (SDQ) ^10^, Conflict and Behavioral Questionnaire (CBQ) ^11^, Pediatric Quality of Life Inventory (PedsQL – Generic Core) ^12^, and the Glasgow Outcome Scale Extended (GOS-E) Pediatric Revision ^13^. Parental distress was measured with the Brief Symptom Inventory (BSI-18) ^14^.

The cognitive battery included tests of premorbid cognitive ability (Wide Range Achievement Test [WRAT4]) ^15^, a shortened measure of effort (Test of Memory Malingering [TOMMe10]) ^16^, and selected tests from the Delis-Kaplan Executive Function System (D-KEFS) ^17^, the Hopkins Verbal Learning Test-Revised (HVLT), and Wechsler Intelligence Scales depending on initial age at assessment. Specifically, the Wechsler Adult Intelligence Scale–IV (WAIS-IV) ^18^ was used for participants 16-18 years old at enrollment whereas the Wechsler Intelligence Scale for Children–V (WISC-V) ^19^ was used for participants 8-15 years old at enrollment. Composite measures of attention (DKEFS color-word interference conditions 1-3), processing speed (WAIS-IV/WISC-V digit symbol coding and symbol search), working memory (WISC-V/WAIS-IV digit span backwards trial), executive function (DKEFS trail making test condition 4, verbal fluency, color-word interference condition 4) and long-term memory recall (HVLT Delay) were compiled to create specific cognitive domains by averaging t-scores from individual tests.

Categorical representations of loss of consciousness (LOC) and post-traumatic amnesia (PTA) were used due to their superior psychometric properties relative to continuous ratings ^1^. The PCSI was also modified in the following ways with author permission: 1) the version of the PCSI for 13-18-year-old participants was also utilized for 12-year-old participants and 2) all references to an injury were removed from both questionnaires and instructions to avoid reporting bias in HC. The retrospective (i.e., one month prior to initial visit) and V1 (day of initial visit) reporting instructions were also specified in the revised version. All summary scores from the PCSI were normalized into percentage values given that different scales were administered to older (ages 12-18) and younger children (ages 8-11).

*Blood-based Biomarker Analyses*

During the course of data collection, Quanterix updated their V2 kit for the pTau-181 assay to Version2.1. A total of 53.5% samples (39/99; 39.4% pmTBI; 53/73; 72.6% HC) were processed using the Version2 kit. Resulting concentration values were transformed to be compatible with the newer Version.2.1 kits using the following formula recommended by Quanterix ^20^.

Y = ((1.651 - 8993) / (1 + (X/1459)^0.9145)) + 8993

Where

Y = converted Version2.1

X = original Version2 value

*Results*

UCH-L1 group comparisons and secondary analyses were run with (main analysis) and without (supplemental analysis) participants who exhibited high COV values. UCH-L1 supplemental analysis excluding participants with high COV data indicated a main effect of visit (*Wald-χ^2^*=6.53; *p=*0.011; V1>V2) and a significant negative correlation with days post injury (DPI; r=-0.55; p=0.001), similar to the main analysis. Similarly, there were no significant effects of PTA/LOC for UCH-L1 levels in the reduced supplemental sample.

**Supplementary Table S1:** Primary and secondary clinical and neurocognitive measures.

| **Instrument** | **Measured domain** | **Status** | **Rater** | **Visit** |
| --- | --- | --- | --- | --- |
| **Demographics** | | | | |
| NewMAP TBI | Self-reported TBI history | Secondary | C & P | R, V1, V2 |
| Tanner Stage of Development | Pubertal development | Secondary | C | V1 & V2 |
| ASSIST | Use of alcohol and other drugs | Secondary | C | V1 & V2 |
| BSI-18 | Parental psychopathology | Secondary | P | V1 & V2 |
| **Clinical Domain** | | | | |
| PCSI | Post-concussive symptoms | Primary | C | R, V1, V2 |
| PROMIS-Sleep | Sleep disturbance | Secondary | C | R, V1, V2 |
| PROMIS-Anxiety | Anxiety symptoms | Secondary | C | R, V1, V2 |
| PROMIS-Depression | Depressive symptoms | Secondary | C | R, V1, V2 |
| Pain scale | Pain | Secondary | C | R, V1, V2 |
| HIT-6 | Headache symptoms | Secondary | C | R, V1, V2 |
| CBQ | Family conflict | Primary | C | R, V1, V2 |
| SDQ | Behavioral screening for psychological attributes | Secondary | P | R & V2 |
| PedsQL | Health-related quality of life | Primary | C | R & V2 |
| GOS-E | Functional outcome | Secondary | C & P | V1 & V2 |
| **Cognitive Domain** | | | | |
| TOMMe10 | Measure of effort | Secondary | C | V1 & V2 |
| WRAT-4 | Premorbid reading ability | Secondary | C | V1 & V2 |
| DKEFS Color-Word interference Cond 1-3 | Attention | Primary | C | V1 & V2 |
| WAIS-IV/WISC-V Coding and Symbol Search | Processing speed | Primary | C | V1 & V2 |
| WISC-V/WAIS-IV Digit Span Backwards | Working memory | Secondary | C | V1 & V2 |
| DKEFS Trail Making Test Cond 4, Verbal Fluency, Color-Word interference Cond 4 | Executive function | Secondary | C | V1 & V2 |
| HVLT Delayed Recall | Long Term Memory Recall | Secondary | C | V1 & V2 |

Notes: Instrument-- NewMAP TBI: New Mexico Assessment of Pediatric TBI, ASSIST: The Alcohol, Smoking and Substance Involvement Screening Test, BSI: Brief Symptom Inventory-18, PCSI: Post-Concussion Symptom Inventory, PROMIS: Patient-Reported Outcomes Measurement Information System, HIT-6: Headache Impact Test, CBQ: Conflict Behavior Questionnaire, SDQ: Strengths and Difficulties Questionnaire, PedsQL: Pediatric Quality of Life Inventory, GOS-E: Glasgow Outcome Scale Extended, TOMMe10: Test of Memory Malingering, WRAT-4: Wide Range Achievement Test, DKEFS: Delis-Kaplan Executive Function System, WAIS-IV: Wechsler Adult Intelligence Scale–IV, WISC-V: Wechsler Intelligence Scale for Children–V, HVLT: Hopkins Verbal Learning Test; Rater—C: child, P: parent; Visit—R: retrospective, V1: Visit 1 (~7 days post-injury), V2: Visit 2 (~4 months post-injury).

**Supplementary Table S2**: Additional summary data for all biomarkers.

|  | LOD (pg/mL) | Range (pg/mL) | LLOQ (pg/mL) | COV>30% or Missing (V1)* | COV>30% or Missing (V2)* | Analysis Sample Size (V1) | Analysis Sample Size (V2) |
| --- | --- | --- | --- | --- | --- | --- | --- |
| NFL | 0.104 | 0.025-0.276 | 0.241 | 2.0% | 1.4% | 100 | 71 |
| GFAP | 0.221 | 0.042-0.481 | 0.467 | 3.0% | 1.4% | 100 | 71 |
| UCH-L1 | 1.90 | 0.855-3.17 | 9.38 | 47.50% | 36.6% | 100 | 71 |
| Tau | 0.024 | 0.007-0.059 | 0.053 | 2.0% | 1.4% | 100 | 71 |
| pTau (V2) | 0.028 | 0.019-0.052 | 0.085 | 2.2% | 0% | 46 | 34 |
| pTau (V2.1) | 0.620 | 0.084-1.211 | 2.00 | 1.9% | 0% | 54 | 37 |

Notes: LOD=Limit of Detection; LLOQ=Lower Limit of Quantification; COV>30% = the percentage of samples with coefficient of variation greater than 30%. LOD, Range and LLOQ obtained from Quanterix website (data sheets). There were no samples for any of the protein biomarkers that were below LOD. A total of 26 UCH-L1 samples were below the LLOQ. There were two assays used for pTau181 calculations with different LOD & LLOQ values. pTau data were transformed using recommended formula from the manufacturer. *The asterisk denotes that a minority of cases were missing CV values due to a missing duplicate analysis on the Quanterix platform.

**References**

1 Hergert, D. C. *et al.* Test-Retest Reliability of a Semi-Structured Interview to Aid in Pediatric Traumatic Brain Injury Diagnosis. *J Int.Neuropsychol.Soc.* **28**, 687-699 (2022). <https://doi.org:10.1017/S1355617721000928>

2 Group, W. The alcohol, smoking and substance involvement screening test (ASSIST): development, reliability and feasibility. *Addiction* **97**, 1183-1194 (2002).

3 Gioia, G. A., Collins, M. & Isquith, P. K. Improving identification and diagnosis of mild traumatic brain injury with evidence: psychometric support for the acute concussion evaluation. *J.Head Trauma Rehabil.* **23**, 230-242 (2008). <https://doi.org:10.1097/01.HTR.0000327255.38881.ca>

4 Gioia, G. A., Schneider, J. C., Vaughan, C. G. & Isquith, P. K. Which symptom assessments and approaches are uniquely appropriate for paediatric concussion? *Br.J.Sports Med.* **43 Suppl 1**, i13-i22 (2009). <https://doi.org:10.1136/bjsm.2009.058255>

5 Buysse, D. J. *et al.* Development and validation of patient-reported outcome measures for sleep disturbance and sleep-related impairments. *Sleep* **33**, 781-792 (2010).

6 Pilkonis, P. A. *et al.* Item banks for measuring emotional distress from the Patient-Reported Outcomes Measurement Information System (PROMIS(R)): depression, anxiety, and anger. *Assessment.* **18**, 263-283 (2011).

7 Farrar, J. T., Young, J. P., Jr., LaMoreaux, L., Werth, J. L. & Poole, R. M. Clinical importance of changes in chronic pain intensity measured on an 11-point numerical pain rating scale. *Pain* **94**, 149-158 (2001).

8 Kriz, P. K. *et al.* Physical Maturity and Concussion Symptom Duration among Adolescent Ice Hockey Players. *J.Pediatr.* **171**, 234-239 (2016).

9 Kosinski, M. *et al.* A six-item short-form survey for measuring headache impact: the HIT-6. *Qual.Life Res.* **12**, 963-974 (2003).

10 Goodman, R. The Strengths and Difficulties Questionnaire: a research note. *J.Child Psychol.Psychiatry* **38**, 581-586 (1997).

11 Prinz, R. J., Foster, S., Kent, R. N. & O'Leary, K. D. Multivariate assessment of conflict in distressed and nondistressed mother-adolescent dyads. *J.Appl.Behav.Anal.* **12**, 691-700 (1979).

12 Varni, J. W., Seid, M. & Rode, C. A. The PedsQL: measurement model for the pediatric quality of life inventory. *Med.Care* **37**, 126-139 (1999).

13 Beers, S. R. *et al.* Validity of a pediatric version of the Glasgow Outcome Scale-Extended. *J.Neurotrauma* **29**, 1126-1139 (2012).

14 Derogatis, L. R. & Fitzpatrick, M. The SCL-90-R, the Brief Symptom Inventory (BSI), and the BSI-18. (2004).

15 Wilkinson, G. S. & Robertson, G. J. *WRAT 4: Wide range achievement test; professional manual*. (Psychological Assessment Resources, Incorporated, 2006).

16 Denning, J. H. The efficiency and accuracy of the Test of Memory Malingering trial 1, errors on the first 10 items of the test of memory malingering, and five embedded measures in predicting invalid test performance. *Arch.Clin.Neuropsychol.* **27**, 417-432 (2012).

17 Delis, D. C., Kaplan, E. & Kramer, J. H. *Delis-Kaplan executive function system (D-KEFS)*. (Psychological Corporation, 2001).

18 Wechsler, D. *Wechsler adult intelligence scale-fourth*. (San Antonio: Pearson, 2008).

19 Wechsler, D. (Bloomington, MN: Pearson Clinical Assessment, 2014).

20 Simoa® pTau-181 Advantage V2.1 Assay. (Quanterix Corporation, 2022).
